# Low-Intensity Physical Exercise Decreases Inflammation and Joint Damage in the Preclinical Phase of a Rheumatoid Arthritis Murine Model

**DOI:** 10.3390/biom13030488

**Published:** 2023-03-07

**Authors:** Susana Aideé González-Chávez, Salma Marcela López-Loeza, Samara Acosta-Jiménez, Rubén Cuevas-Martínez, César Pacheco-Silva, Eduardo Chaparro-Barrera, César Pacheco-Tena

**Affiliations:** Laboratorio PABIOM, Facultad de Medicina y Ciencias Biomédicas, Universidad Autónoma de Chihuahua, Chihuahua 31125, Mexico

**Keywords:** physical exercise, preclinical arthritis, at-risk arthritis, inflammation, joint damage, microarray

## Abstract

Lifestyle modifications in preclinical Rheumatoid Arthritis (RA) could delay the ongoing pathogenic immune processes and potentially prevent its onset. Physical exercise (PE) benefits RA patients; however, its impact in reducing the risk of developing RA has scarcely been studied. The objective was to describe the effects of low-intensity PE applied at the disease’s preclinical phase on the joints of DBA/1 mice with collagen-induced arthritis (CIA). Twelve mice with CIA were randomly distributed into two groups: the CIA-Ex group, which undertook treadmill PE, and the CIA-NoEx, which was not exercised. The effects of PE were evaluated through clinical, histological, transcriptomics, and immunodetection analyses in the mice’s hind paws. The CIA-Ex group showed lower joint inflammation and damage and a decreased expression of RA-related genes (*Tnf Il2*, *Il10*, *Il12a*, *IL23a*, and *Tgfb1*) and signaling pathways (Cytokines, Chemokines, JAK-STAT, MAPK, NF-kappa B, TNF, and TGF-beta). TNF-α expression was decreased by PE in the inflamed joints. Low-intensity PE in pre-arthritic CIA reduced the severity through joint down-expression of proinflammatory genes and proteins. Knowledge on the underlying mechanisms of PE in preclinical arthritis and its impact on reducing the risk of developing RA is still needed.

## 1. Introduction

Rheumatoid arthritis (RA) is an inflammatory autoimmune disease whose pathogenesis is complex and yet incompletely understood [1]. RA has a preclinical, asymptomatic phase in which environmental stimuli interact with a genetically predisposed host. Eventually, these interactions result in breach of tolerance in sites such as the mucosal surfaces, leading to the initiation, maturation, and amplification of autoimmunity outside the joint. Subsequently, active elements of the innate and adaptative immune response access the synovium, resulting in overt synovitis that is clinically detectable. The period before clinical inflammatory arthritis is designated preclinical RA in those individuals who have progressed to a clinical diagnosis of RA, and an at-risk status in those who exhibit predictive biomarkers of RA but have not developed inflammatory arthritis [2,3,4,5].

A better comprehension of the preclinical pathophysiological process contributes to identifying critical mediators of the disease and may allow the establishment of lifestyle modifications and specific therapies in the early preclinical stages that can delay the ongoing pathogenic immune processes and potentially prevent RA onset [6]. For example, a recent systematic review demonstrated that the early treatment of at-risk individuals might effectively delay RA onset, thereby decreasing disease-related limitations in individuals in the preclinical phase of RA [7]. On the other hand, non-pharmacological preventive strategies, such as the modification of behavioral RA-risk factors (smoking, obesity, low physical activity (PA), low-quality diet, and poor dental hygiene) among at-risk individuals, may decrease RA risk [5,8].

The beneficial effects of PA and physical exercise (PE) on the clinical, metabolic, and cardiorespiratory features in patients with established RA have been widely documented [9]; indeed, PA is considered part of the comprehensive management of patients [10,11]. Nevertheless, studies evaluating the effects on the risk of developing RA are scarce, and evidence from observational studies is not entirely consistent [12,13]. Furthermore, the heterogeneity of the designs of the studies evaluating PE in RA, aside from the high risk of bias, precludes conclusive results; therefore, further research is still necessary [14].

The effects of PE on joint biology, including the inflammatory process and structural damage, have not been sufficiently explored, partly due to the inconvenience of repeated biopsies from human joints in PE trials. In this regard, animal models of RA provide a valuable tool for exploring this field. We have recently reviewed the topic [15], finding that, in animal models of RA, PE can potentially exacerbate the joint inflammatory process; however, the information is inconclusive as some studies have also shown a beneficial effect. In our review, we described how the differential metabolic and immune effects of PE are dependent on numerous extent variables, including the animal model (species, strain, and type of arthritis induction), the type of PE (aerobic or anaerobic), the intensity (low-, moderate- or high-intensity), the time of intervention (morning and night), the use of stimulus (voluntary and forced), and the duration (acute and chronic) [15].

The exploration of the impact of PE, in any of its modalities and variants, in the preclinical phase of joint inflammation in animal models of RA is practically nil. Therefore, we aimed to evaluate the effect of low-intensity PE in preclinical collagen-induced arthritis (CIA) in DBA/1 mice. The joint biology was evaluated through transcriptomic, clinical, and histopathological analyses.

## 2. Materials and Methods

The effects of low-intensity treadmill PE on joint biology in the preclinical CIA in DBA/1 mice were evaluated by comparing exercised and non-exercised mice. In addition, clinical and histological severity, transcriptome modifications, and protein expression were analyzed through histopathology, DNA microarray, and reverse transcription (RT)-quantitative polymerase chain reaction (qPCR).

### 2.1. Animals and Study Groups

The study included 16 male DBA/1 mice aged 11–12 weeks from the Faculty of Medicine and Biomedical Sciences of the Autonomous University of Chihuahua, in which CIA was induced. Mice were randomly distributed into two groups with eight mice each: (1) the CIA-Ex group, which received PE from day 14 (one day after the second collagen injection), and (2) the CIA-NoEx group, which did not exercise and was the control group. The sample size was calculated with the formula for comparison between two groups for quantitative data, with a type I error of 5%, statistic power of 80%, and standard deviation (SD) and effect size from our previous research evaluating the effect of exercise in arthritis rodent models [16,17].

Mice from two groups were kept in the same building under controlled luminosity (12 h light/12 h dark) and temperature (23 ± 2 °C) and received food and water ad libitum. Mice were monitored by the researchers and the veterinarian throughout the entire research, including the PE routines. We established a priori that the exercise session should be stopped if mice have an accidental injury, fatigue, unexpected adverse effects, behavioral issues, or poor performance caused by the animal’s unwillingness to exercise. Additionally, we established that an animal should be removed if it becomes permanently unable to perform the exercise and requires excessive motivation to exercise. However, no mouse removal was necessary, and all analyses included eight mice per group. This study complied with the Official Mexican Standard NOM-062-ZOO-1999 technical specifications for producing, caring for, and using laboratory animals. The research was approved by the Ethics Committee and Institutional Animal Care and Use Committee (IACUC), with the ID number: CI-036-20.

### 2.2. Arthritis Induction

The CIA was performed as described by Brand et al. [18] in 11–12-week-old mice. Under isoflurane anesthesia, mice were intradermally injected in the tail base with a suspension containing 0.1 mg of type II bovine collagen emulsified with complete Freund’s adjuvant (day 0). A second injection was applied on day 14 using Freund’s incomplete adjuvant and the same quantity of collagen. Mice were sacrificed once they completed the PE intervention. The incidence and clinical score of arthritis were evaluated using the semiquantitative scale, i.e., 0: no evidence of erythema and swelling; 1: erythema and mild swelling confined to the tarsals or ankle joint; 2: erythema and mild swelling extending from the ankle to the tarsals; 3: erythema and moderate swelling extending from the ankle to metatarsal joints; and 4: erythema and severe swelling encompass the ankle, foot, and digits. The total score per mouse was obtained by adding the score of the four limbs.

### 2.3. Treadmill Physical Exercise Intervention

**Figure 1:**
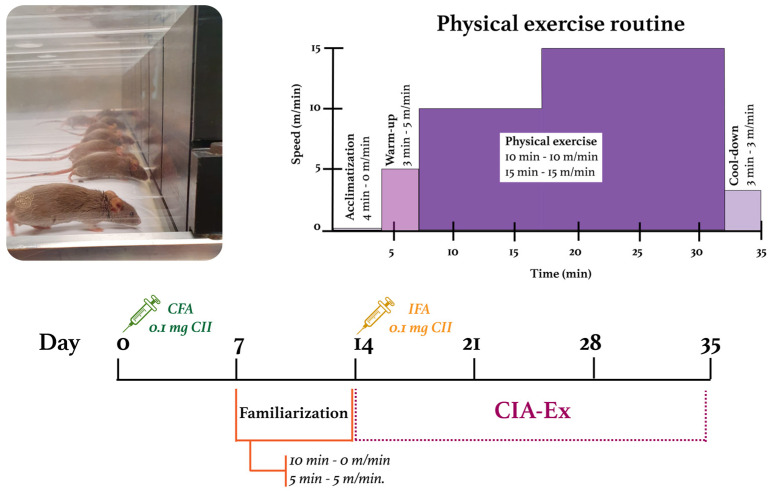
Experimental design and characteristics of the treadmill physical exercise routine. CFA: Complete Freund’s Adjuvant; IFA: Incomplete Freund’s Adjuvant; CII: Collagen type II.

The PE intervention was performed on a custom-designed and built treadmill compliant with the American Physiological Society recommendations. Specific times and speeds were programmed using the software MRLabEx (Figure 1).

For mice in the experimental group, a one-week familiarization period was included to minimize the psychological stress of the mice by promoting visual/olfactory and sound/motion adaptation to the treadmill. Mice were placed on the treadmill daily for 15 min: 10 min on the switched-off and 5 min walking at 5 m/min. Familiarization occurred between the first and second collagen injections (Figure 1). 

After the familiarization period, the speed at which the mice would perform the low-intensity exercise (30% and 50% of their maximum capacity) was calculated. Treadmill-running performance was evaluated through a treadmill exhaustion test. Mice were placed on the band and ran for five minutes at 5 m/min as a warm-up. After this time, the speed increased by 3 m/min every 2 min until the mice were exhausted. The maximum PE capacity (100%) was defined as the maximum speed reached by each mouse; then, the speed’s mean and SD were obtained per group. DBA/1 mice’s maximum PE capacity resulted in 32.9 ± 2.9 m/min, so 30% and 50% of their capacity were defined as 10 m/min and 15 m/min, respectively.

Mice from the CIA-Ex group were exercised for three weeks with a frequency of five 35 min sessions per week (Monday to Friday). The PE session included the phases of (1) acclimatization: mice were placed for 4 min on the switched-off treadmill; (2) warm-up: mice walked for 3 min at 5 m/min; (3) PE: mice ran for 10 min at 10 m/min, followed by 15 min at 15 m/min; and (4) cool-down: mice walked for 3 min at 3 m/min. Treadmill PE intervention started when the clinical arthritis score was ≤1, the day after the second collagen injection. Mice from both groups were euthanized with isoflurane on day 35 (Figure 1).

### 2.4. DNA Microarray and Bioinformatic Analysis

RNA was obtained from tarsal bones and joints, including ligaments. Tissues were disrupted in liquid nitrogen using a biopulverizer. Total RNA was purified using the RNeasy^®^ Mini Kit (Qiagen, Hilden, Germany), following the manufacturer’s protocol. The RNA quantity and quality were verified in the Qubit4 fluorometer (ThermoFisher Scientific, Waltham, MA USA). The RNA of each mouse in each study group was mixed in equimolar amounts to conform pools used in the DNA microarray.

The microarray was carried out at the Institute of Cellular Physiology, Autonomous University of Mexico (UNAM), Mexico. DNA microarray was performed to compare the CIA-Ex group with respect to the CIA-NoEx group. Briefly, the RT-PCR was performed and the resulting complementary DNA (cDNA) from the CIA-Ex group was labeled with Cy5, while the cDNA from the CIA-NoEx group was labeled with Cy3. Hybridization was performed using the M22K_01 (UNAM, Mexico City, Mexico) chip containing 22,000 genes from the mouse genome. The scan and signal acquisition were developed using the ScanArray 4000 (Packard BioChips Technologies, Billerica, MA, USA). The analysis of the microarray scanning was carried out using GenArise Microarray Analysis Tool software (UNAM), and the lists of differentially expressed genes (DEGs) (Z-score ≥ 1.5 SD) were obtained [19]. The microarray dataset was registered in the Gene Expression Omnibus (GEO) of the National Center for Biotechnology Information (NCBI) database with the accession number GSE212262. 

The lists of DEGs by PE were further analyzed in DAVID Bioinformatics Resources 6.8 (https://david.ncifcrf.gov/, accessed on 1 January 2023), an open-resource platform that classifies genes list into functional biological processes and KEGG (Kyoto Encyclopedia of Genes and Genomes) signaling pathways [20]. Furthermore, the STRING database 11.5 (https://string-db.org/, accessed on 1 January 2023) was used to obtain the analysis and integration of direct and indirect protein–protein interactions (PPI) centered on the functional association [21]. The DEGs identified in the microarray were loaded, and the interactions with minimal confidence (interaction score > 0.4) were selected. The PPI network was more thoroughly analyzed to obtain primary clusters of sub-networks using the Cytoscape software v3.9.1 with the Molecular Complex Detection (MCODE) complement (node score cutoff = 0.6) [22,23]. The genes of the two primary clusters were loaded in the STRING database to show the PPI-associated KEGG pathways; genes of relevance in arthritis were identified in different colors.

### 2.5. Histopathological Analysis

The hind paws were dissected and fixed in 10% phosphate-buffered formalin for 48 h, and were then demineralized using 5% nitric acid for 48 h, dehydrated in graded ethanol, and embedded in paraffin. Sections of 5 μm in thickness were obtained and placed on adhesive-coated glass slides. Histological assessment was carried out using hematoxylin and eosin (H&E) staining. The images were acquired using a digital camera coupled to the optical microscope. The influence of PE on tarsal joint structures was evaluated in three slices of each sample, using the semiquantitative scale of 0, absent; 1, mild; 2, moderate; or 3, severe to describe inflammatory infiltrate, synovial hyperplasia, cartilage damage, and bone erosion in the tarsal joints. The mean score was calculated for each group. The histological parameters were the primary outcome measured.

IHC analysis was performed with a specific antibody against tumor necrosis factor (TNF)-α (sc-52746, Santa Cruz Biotechnology, Dallas, TX, USA). Tissue sections were deparaffinized in xylene and dehydrated in descending concentrations of ethanol until water. Antigen retrieval was carried out using 0.05% trypsin (T1426-250 mg, SIGMA Life Science, St. Louis, MO, USA) for 20 min at 37 °C, and then tissues were treated with 0.2% Triton-X100 (Bio-Rad, Hercules, CA, USA). After blocking with 10% bovine serum albumin (BSA) (Sigma Life Science, St. Louis, MO, USA) for 30 min at 37 °C in a humidified chamber, tissues were treated with hydrogen peroxide for 10 min at room temperature to remove endogenous peroxidase activity. Tissues were incubated with the primary antibody diluted 1:1000 in 1% BSA at 4 °C overnight. The corresponding isotype’s biotin-streptavidin-conjugated secondary antibody (Jackson ImmunoResearch Laboratories, Inc., West Grove, PA, USA) was used in a 1:400 dilution. Immunodetection was carried out using Pierce^®^ streptavidin horseradish peroxidase-conjugated (Jackson ImmunoResearch Laboratories, Inc., West Grove, PA, USA) and Diaminobenzidine (DAB) (D4293-50SET, SIGMA-ALDRICH Co., St. Louis, MO, USA) as the chromogen. The primary antibody was replaced with PBS buffer to establish a negative control. Images were acquired using a digital camera (AmScope MU1803, Irvine, CA, USA) coupled with an optical microscope (AxioStar Plus, Carl Zeiss, Berlin, Germany), taking at least 20 microscopic fields from each study subject. The expression of each marker was quantified with the ImageJ program and the IHC toolbox. The DAB color was extracted from each image, and the maximum and mean gray values were obtained. Each image’s optical density (OD) was obtained with log10 (maximum gray value/mean gray value). The OD means and SD were calculated and graphed per study group.

### 2.6. Inflammatory Cytokines RNA Quantification by RT-qPCR

RNA quantification was performed through RT-qPCR for *Tnf*, *interleukin (Il)2*, *Il6*, *Il10*, *Il12a*, *IL23a*, *transforming growth factor* (*Tgfb1*), and *Janus kinase* (*Jak*)*3*, with the primers sets listed in Table 1. The *ribosomal protein L* (*Rpl*)*13* was used as the reference gene [24]. The cDNA was synthesized from total RNA (1µg) by RT using the SupeScript^®^ III First-Strand Synthesis System (Invitrogen by ThermoFisher Scientific) with random hexamer primers, and was then diluted to 100 µL. For each gene, 3 µL of individual cDNA was used for qPCR using the Radiant™ SYBER Green Hi-ROX qPCR kit (Radiant). Data were collected in real time during the elongation step of each cycle, using the Quant Studio 3 PCR System (ThermoFisher Scientific). Each cDNA sample was analyzed in triplicate. The relative quantification was performed using the ΔΔCt method.

### 2.7. Statistical Analysis

The bioinformatics analysis of the microarray data included their statistical analysis. In DAVID, Fisher’s exact test is adopted to measure gene enrichment in annotation terms. Fisher’s Exact *p*-values are computed by summing probabilities *p* over defined sets of tables (Prob = ∑Ap) [20]. In the STRING database, the PPI enrichment *p*-value indicates that the nodes are not random and that the observed number of edges is significant; for the associated-KEGG pathways, the false discovery rate (FDR) is defined as FDR = E (V/R|R > 0) P (R > 0) [25]. In Cytoscape-MCODE, the complex score is defined as the product of the complex subgraph, C = (V, E), density, and the number of vertices in the complex subgraph (DC × |V|) [23].

For clinical and histological variables and IHC and RT-qPCR measures, statistical analysis was made in SPSS statistics v22 software (SPSS Science Inc., Chicago, IL, USA). The Shapiro–Wilk and Kolmogorov–Smirnov tests were used to determine the data normality. Measures of central tendency and dispersion were estimated for each variable, and a Mann–Whitney U test was used to compare the effect of PE in the CIA-Ex group compared with the CIA-NoEx control group. Differences were considered significant when *p* ≤ 0.05.

## 3. Results

**Figure 2:**
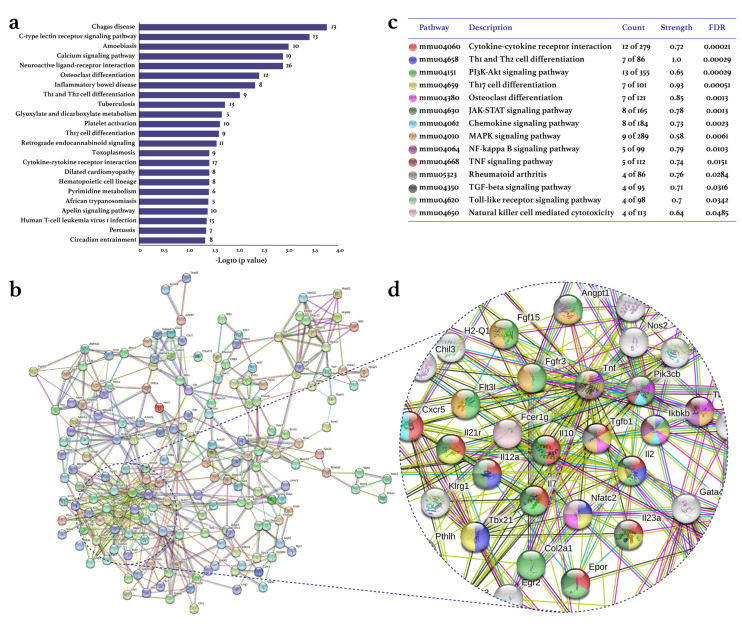
Effect of physical exercise on transcriptomic down-expression in tarsal joints of mice with collagen-induced arthritis. The lists of the down-expressed genes in the CIA-Ex mice with respect to control (Z-score ≥ 1.5 SD) were analyzed on DAVID bioinformatics resource, STRING database, and Cytoscape software (n = 8 mice per group). (**a**) The associated KEGG pathways (*p* ≤ 0.05) in DAVID are shown in the bars that indicate the -Log10 (*p*-value) and the number of genes on the right side of each bar. (**b**) The primary clusters of sub-networks found with the Molecular Complex Detection (MCODE) complement (cutoff = 0.4) to obtain the protein–protein interaction (PPI) network. (**c**) KEGG signaling pathways relevant in arthritis. (**d**) Amplification of the nodes with the most significant interaction in the selected pathways. FDR: false discovery rate.

**Figure 3:**
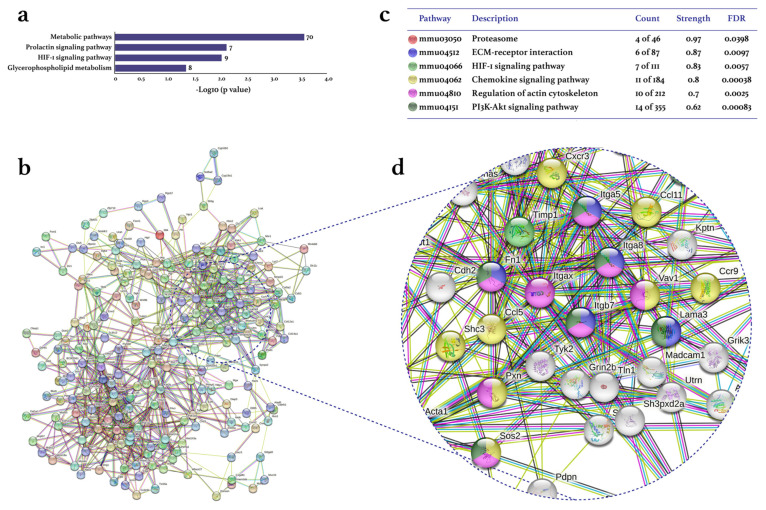
Effect of physical exercise on transcriptomic up-expression in tarsal joints of mice with collagen-induced arthritis. The lists of the up-expressed genes in the CIA-Ex mice with respect to control (Z-score ≥ 1.5 SD) were analyzed on DAVID bioinformatics resource, STRING database, and Cytoscape software (n = 8 mice per group). (**a**) The associated KEGG pathways (*p* ≤ 0.05) in DAVID are shown in the bars that indicate the -Log10 (*p*-value) and the number of genes on the right side of each bar. (**b**) The primary clusters of sub-networks found with the Molecular Complex Detection (MCODE) complement (cutoff = 0.4) to obtain the protein–protein interaction (PPI) network. (**c**) KEGG signaling pathways relevant in arthritis. (**d**) Amplification of the nodes with the most significant interaction in the selected pathways. FDR: false discovery rate.

Treadmill PE differentially expressed 2097 genes (1126 up/971 down) in the CIA-Ex group if compared to the CIA-NoEx. The bioinformatics analysis in the DAVID platform showed that the DEGs were significantly associated with 27 KEGG pathways (four up/23 down). The down-expressed KEGG pathways included those related to immune/inflammatory processes, such as Th1 and Th2 cell differentiation, Th17 cell differentiation, cytokine-cytokine receptor interaction and osteoclast differentiation (Figure 2a and Table S1). The up-expressed KEGG pathways included metabolic pathways and the hypoxia-inducible factor (HIF)-1 signaling pathway (Figure 3a and Table S1). 

The analyses in the STRING database and Cytoscape-MCODE software resulted in the PPI networks shown in Figure 2b and Figure 3b. The PPI from down-regulated genes resulted in 179 nodes and 572 edges, while from up-regulated genes in 207 nodes and 761 edges. PE down-regulated genes of several KEGG pathways related to immune/inflammatory processes, including cytokine–cytokine receptor interaction, Th1 and Th2 cell differentiation, phosphoinositide 3-kinase (PI3K)-Akt, Th17 cell differentiation, osteoclast differentiation, JAK-STAT, chemokines, mitogen-activated protein kinase (MAPK), nuclear factor (NF-)kappa B, TNF, TGF β, Toll-like receptor, and RA signaling pathways (Figure 2c). This PPI network highlighted the down-expression of the genes *Tnf*, *Tgfb1*, *Il2, Il10*, *ll2a*, and *Il23* (Figure 2d). On the other hand, the up-expressed genes were only associated with the RA-related KEGG pathways of HIF-1, chemokine, and PI3K-Akt (Figure 3c,d).

**Figure 4:**
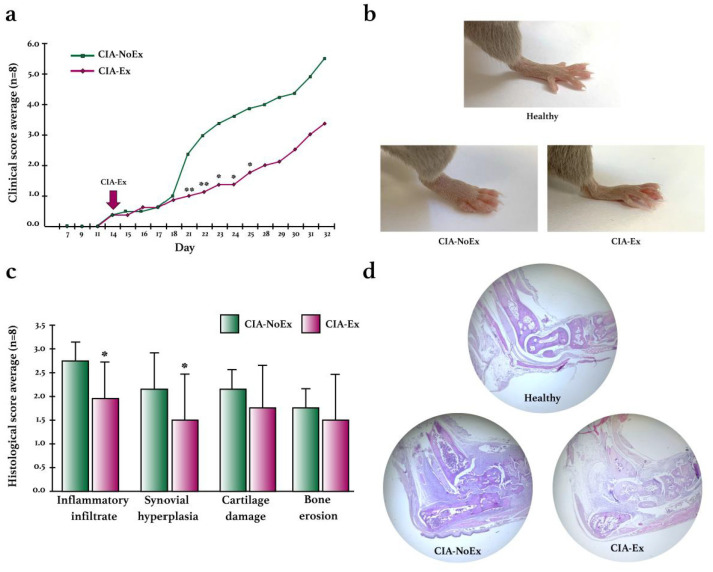
Clinical and histological effects of treadmill physical exercise in the preclinical collagen-induced arthritis in DBA/1 mice. (**a**) Evaluation of the clinical course of arthritis. Erythema and swelling were evaluated using the semiquantitative scale from 0 to 4 in each paw and the sum of the four paws per mouse was obtained. The mean score and standard deviation were calculated and graphed for each group during the experimental intervention. (**b**) Representative images of the clinical manifestation of CIA in control and exercised mice. (**c**) Histological evaluation in the hind paws by H&E staining. Inflammatory infiltrates, synovial hyperplasia, cartilage damage, and bone erosions were evaluated using the semiquantitative scale from 0 to 4. The mean score and standard deviation were calculated and graphed for each group. (**d**) Representative images of the histological findings in the hind paws using H&E staining. The Mann–Whitney U test was used to determine statistically significant differences when *p* < 0.05. * *p* ≤ 0.05; ** *p* ≤ 0.01.

The effect of PE was also evaluated on clinical and histological arthritis (Figure 4). In the CIA-Ex group, the progression of swelling and erythema was lower than in the control group, reaching statistical differences at week 4 (Figure 4a,b). In addition, the histological analysis at the end of the experiment showed that PE significantly decreased inflammatory infiltrate and synovial hyperplasia (Figure 4c,d).

**Figure 5:**
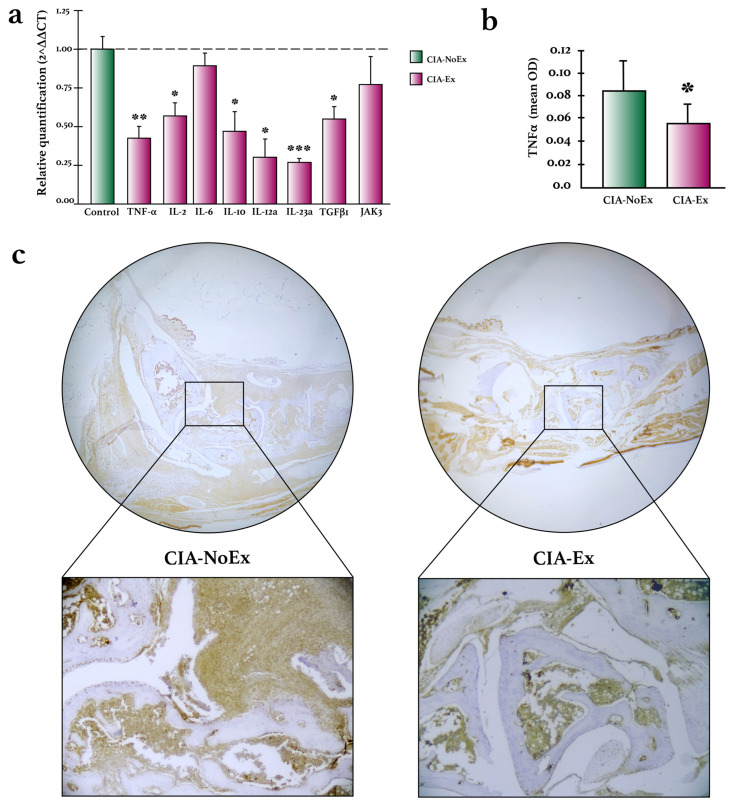
Effects of treadmill physical exercise on inflammatory cytokine expression in the preclinical collagen-induced arthritis in DBA/1 mice. (**a**) Relative quantification of inflammatory cytokines on hind paws joints. RNA expression was determined by RT-qPCR relative quantification through the ΔΔCt method using the housekeeping gene RPL13A. (**b**) TNF-α expression was quantified with the ImageJ program and the IHC toolbox in at least 20 microscopic field images from each study subject. The DAB color was extracted from each image, and the maximum and mean gray values were obtained. Each image’s optical density (OD) was obtained with log10 (maximum gray value/mean gray value). The OD means and standard deviations were calculated and graphed by the study group. (**c**) Representative images of TNF-α detection in hind paws joints by immunohistochemistry. Immunodetection was carried out using streptavidin-peroxidase conjugated and DAB as the chromogen. The Mann–Whitney U test was used to determine statistically significant differences. * *p* < 0.05; ** *p* < 0.01; *** *p* < 0.001.

The expression of inflammatory cytokines was analyzed by RT-qPCR and IHC (Figure 5). Compared to the CIA-NoEx control group, the relative expression of *Tnf*, *Il2*, *Il10*, *Il12a*, *ILl23a*, and *Tgfb1* was significantly lower in the CIA-Ex group (Figure 5a). Additionally, the expression of TNF-α protein was significantly lower in the hind paws of the CIA-Ex group (Figure 5b,c).

## 4. Discussion

Our current understanding of RA pathogenesis states that environmental factors induce specific post-translational modifications in genetically susceptible individuals, which trigger the pathological activation of the immune system and lead to disease onset. Consequently, research directed at the preclinical phases of RA is increasing since it is considered an opportune timeframe for preventive interventions, including pharmacological approaches and potential lifestyle modifications. 

PE is a proven and widely recommended strategy to preserve the health of the general population and patients with RA. Under physiological conditions, PE induces several intracellular signaling pathways that result in cellular endurance and adaptation in the musculoskeletal system [26,27]. Moreover, PE also influences the regulation of innate immunity and inflammation [28,29].

The precise role of PE in the joints of patients with RA is rarely reported in humans due to the unavailability of joint tissue. Most studies in animal models of RA dealing with PE focus on the differential effect of diverse types and doses of PE [30,31,32,33], while its impact on the preclinical phases has scarcely been explored. Animal models of RA demonstrate that PE influences joint pathological processes once arthritis is established; however, its effects are not conclusive [15]. Although positive outcomes of PE have been described, including the decrease in arthritis severity [32,34] and the down-expression of inflammatory genes and pathways [16], the exacerbation of joint damage by PE has also been reported [17,31,35]. This relation between PE and the influence in the arthritis course may differ in the pre-arthritic stage.

In the present study, the low-intensity PE intervention began on the day of the second collagen injection, when joint inflammation was practically absent in the mice. The clinical evolution was significantly milder in the exercise group, and histologically less joint damage was also found at the end of the experiment. Microarray transcriptomic analysis revealed that PE deregulated genes and signaling pathways crucial to the pathogenesis of RA. Moreover, the down-expression of TNF-α was also confirmed by RT-qPCR and IHC. TNF-α is considered the major inflammatory cytokine in the pathogenesis of RA, and a successful target for biological therapy. TNF-α activates synovial fibroblasts, promotes epidermal hyperplasia, and recruits inflammatory cells [36].

The down-regulation of the Jak-STAT pathway demonstrated in our microarray was also a relevant finding because JAK/STAT plays a crucial role in the inflammatory process of the synovium and bone destruction in RA. Therefore, JAK inhibitors are currently used in patients with moderate to severe RA [37]. Interestingly, a recent study revealed that JAK-STAT signaling is up-regulated in high-risk individuals that developed joint inflammation since the pre-arthritic stage [38].

We also confirmed that low-intensity PE down-expressed genes (*Il2*, *Il10*, *Il12a*, *Il23a*, and *Tgfb1*) and signaling pathways (cytokine–cytokine receptor interaction, Th1 and Th2 cell differentiation, Th17 cell differentiation, and chemokine signaling pathway) that are identified in the phases of maturation and amplification of the systemic immune response that predates RA [2]. The reduction of several cytokines could be due to the overall decrease in the inflammatory process and, therefore, to a reduced involvement of active immune cells. In many cases, however, TNF-α and IL-10 are antagonists since the increased production of TNF-α increases the production of IL-10, creating a negative-feed loop that results in the subsequent reduction of TNF-α [39,40,41]; IL-10 is considered as an anti-inflammatory cytokine, yet that could be an oversimplification [42]. However, several interactions influence the production of IL-10 aside from TNF-α, and most relate to cytokines involved in the Th1/Th2 balance, such as IFN-γ. Under several pathologic scenarios, it has been shown that IL-10 and TNF-α concentrations fluctuate in the same direction [43,44,45] as we observed in our experiment. Interestingly, in most published articles, the effect of PE on IL-10 production shows an upregulation, and therefore PE is considered an anti-inflammatory intervention [46,47,48,49]. Nevertheless, some studies show no significant change [50,51], and in some other cases, as we found in our experiment, a decrease in IL-10 levels after PE has also been described [52,53,54,55,56]. The context in which this influence is explored and the timing after the PE could explain some of this variability. Regarding TGF-β, a key cytokine for tissue remodeling, given the context PE can either increase [57,58,59] or decrease [60,61,62] its production.

Contrary to our findings, Fujii Y. et al. [34], who evaluated the impact of treadmill PE in pre-arthritic and established CIA in DA rats, found that only in the established arthritis did PE inhibit joint destruction, improve bone morphometry and reduce connexin (Cx)43 and TNF-α expression in the synovial membranes. Additionally, Cambré, I. et al. [35], who applied voluntary wheel running one day after the first collagen injection as well as one day after the booster (day 22) in C57BL/6 mice with CIA, found that exercised mice had a marked accelerated onset and significantly more inflammation in the forefoot of the hind paw. Moreover, their microarray analysis of the Achilles tendon showed that voluntary running increased the expression of proinflammatory genes such as *Ccr2*, *Ccl2*, *Cxcl1*, *Mmp3*, *Lira6*, *Icam1*, *Ctsk*, and *Nfatc1*.

The above suggests that, as in the studies evaluating PE in arthritis-induced rodent models, when the PE is applied in the pre-arthritic phases the findings are heterogeneous; its effects possibly depends on the type and intensity of the PE. 

The intensity of the PE is a parameter that directly affects its physiological responses, which has been demonstrated in several studies in animal models. Toti et al. [63] investigated the effect of two different exercise protocols (at 60% or 90% of the maximal running velocity) on the fiber composition and metabolism of two specific muscles of healthy mice. They demonstrated that high-intensity exercise, in addition to metabolic changes consisting of a decrease in blood lactate and body weight, induces an increase in the mitochondrial enzymes and slow fibers. Moreover, this same group of researchers also found that the intensity of the exercise differentially affects the increase in the size of the adrenal gland; they reported a rise of 31.04% for mice that underwent high-intensity PE and 10.08% for mice that underwent low-intensity exercise, and this appeared to be the result of an increase in the area of both the adrenal cortex and adrenal medulla [64].

The application of low-intensity exercise in murine models of inflammatory arthritis, including RA and SpA, once the arthritis is established, has shown chiefly positive effects, whether applying aerobic exercise on a treadmill or in wheel running. Positive effects include the decreased expression of inflammatory cytokines (TNF-α, Cx43 [32,34], *Il2rb*, *Il2ra*, *Il4*, *Il5*, *Il3*, *Cxcl9*, and *Cxcl12*), the infra-expression of inflammatory signaling pathways (chemokine, cytokine-cytokine receptor interaction, complement, coagulation cascade, PI3K-Akt, Jak-STAT, and TLR [16]), the down-expression of markers of pain and disability such as the calcitonin gene-related peptide (CGRP) [65], and the decreased concentration of oxidative stress markers such as thiobarbituric acid reactive substances (TBARS) [66].

Nevertheless, this positive effect of low-intensity exercise cannot be generalized. In our experience, the application of the same low-intensity PE routine to two different rodent models of arthritis resulted in opposite effects. While in proteoglycan-induced arthritis (PGIA) in mice low-intensity PE resulted in a beneficial effect, including less joint destruction [16], in adjuvant-induced arthritis (AIA) in rats the same PE routine exacerbated the destruction process through the over-expression of hypoxia and oxidative stress processes [17]. Similarly, in the experiments carried out by Cambré et al. [31,35] evaluating the effect of voluntary wheel running PE in different models of RA and SpA, it was shown that PE accelerates the arthritis onset and increases its severity at the clinical, histological, and molecular level. The above highlights that, in addition to the intensity of the PE, the type of inflammatory arthritis is also a defining parameter in its effects.

High-intensity exercise, on the other hand, is often associated with an increased risk of joint damage or exacerbation of inflammation that leads to osteoarthritis (OA) [67,68]. However, reports of high-intensity PE in models of inflammatory arthritis (RA and SpA) are practically nil, most likely because the level of joint inflammation (as shown in Figure 4b, below left) prevents the mice from doing this PE without compromising their well-being.

In the present study, low-intensity PE resulted in a protective effect for the arthritis onset and a decrease in its severity; however, according to research carried out in the clinical phases of arthritis, as well as in models of other diseases or health conditions, this effect could not be generalized for moderate- or high-intensity PE. Ideally, these findings should be considered in prescribing PE for at-risk individuals since, considering the heterogeneity of the genetic and clinical presentation of the human disease, as well as the extent of types and intensities of PE, its effects on joint biology cannot be oversimplified.

Finally, we recognize that the design of our study could have been improved by the inclusion of healthy mice that performed the same PE routine, as an additional control. However, we defined the scope of our study as evaluating the effect PE had when applied in arthritis’ preclinical phase, and decided to include only two groups for comparison, both susceptible to developing arthritis. The reason behind this decision was the complexity in the comparison of more than two groups because, in the type of microarrays that we use, only the comparison of two groups is allowed to obtain the list of DEGs; concluding more than one comparison would limit our possibility to derive conclusions. In addition, we considered that the effect of PE in healthy mice had been extensively explored, and we wanted to understand the differences between the pre-arthritic stage selectively and once the inflammation sets in. We recognize that setting our experiment in these two groups can be considered a limitation of our investigation.

## 5. Conclusions

The present study explored the effect of low-intensity PE when applied in the pre-arthritic stage in CIA, demonstrating that, on the joints, PE reduced the severity of clinical and histological arthritis and down-expressed RA-related genes and signaling pathways, suggesting a beneficial effect. However, more research is needed on the types of exercise that can be implemented as a preventative strategy for RA in high-risk individuals.

## Figures and Tables

**Table 1:** Sequences of primers of inflammatory cytokines quantified by RT-qPCR.

Gene	Forward (Sequence 5′–3′)	Reverse (Sequence 5′–3′)
*Tnf*	CCCTCACACTCAGATCATCTTCT	GCTACGACGTGGGCTACAG
*Il2*	TGAGCAGGATGGAGAATTACAGG	GTCCAAGTTCATCTTCTAGGCAC
*Il6*	TAGTCCTTCCTACCCCAATTTCC	TTGGTCCTTAGCCACTCCTTC
*Il10*	GCTCTTACTGACTGGCATGAG	CGCAGCTCTAGGAGCATGTG
*Il12a*	CTGTGCCTTGGTAGCATCTATG	GCAGAGTCTCGCCATTATGATTC
*Il23a*	ATGCTGGATTGCAGAGCAGTA	ACGGGGCACATTATTTTTAGTCT
*Tgfb1*	CTCCCGTGGCTTCTAGTGC	GCCTTAGTTTGGACAGGATCTG
*Jak3*	CCATCACGTTAGACTTTGCCA	GGCGGAGAATATAGGTGCCTG
*Rpl13a*	AGCCTACCAGAAAGTTTGCTTAC	GCTTCTTCTTCCGATAGTGCATC

*Tnf: tumor necrosis factor; Il: interleukin; Tgf: transforming growth factor; Jak: Janus kinase; Rpl: ribosomal protein L.*

## Data Availability

The microarray dataset was registered in the GEO database of the NCBI with the accession number GSE212262.

## Supplementary Materials

The following supporting information can be downloaded at: https://www.mdpi.com/article/10.3390/biom13030488/s1, Table S1: Differentially expressed KEGG signaling pathways by treadmill physical exercise in the preclinical phase of collagen-induced arthritis in DBA/1 mice.
