# Supplementary material for: Low-Intensity Physical Exercise Decreases Inflammation and Joint Damage in the Preclinical Phase of a Rheumatoid Arthritis Murine Model
Source: Biomolecules. 2023 Mar 7;13(3):488. doi: 10.3390/biom13030488 (PMC10046494; doi:10.3390/biom13030488)
Supplement: Supplementary file 1 [file biomolecules-13-00488-s001.zip › biomolecules-2186371-supplementary.pdf]

Supplementary Table S1. Differentially expressed KEGG signaling pathways by treadmill physical exercise in the preclinical phase of collagen-induced arthritis in DBA/1 mice.

**Down-expressed KEGG Pathway (count; *p-value*):** *genes*
**Chagas disease (13; *1.70E-04*):** *Gnai1, Gnaq, Ikbkb, Il10, Il12a, Il2, Mapk13, Nos2, Pik3cb, Plcb1, Serpine1, Tgfb1, Tnf*
**C-type lectin receptor signaling pathway (13; *3.80E-04*):** *Clec4e, Fcer1g, Egr2, Ikbkb, Il10, Il12a, Il2, Il23a, Mapk13, Nfatc2, Pik3cb, Ppp3cc, Tnf*
**Calcium signaling pathway (19; *1.30E-03*):** *Adora2b, Adcy4, Cysltr2, Drd1, Fgf15, Fgfr3, Gnaq, Itpkb, Lhcgr, Ntrk3, Nos2, Plcb1, Plcd3, Phka1, Ppp3cc, Ryr1, Slc8a3, Trhr2, Tnnc2*
**Neuroactive ligand-receptor interaction (26; *1.30E-03*):** *Htr1d, Adora2b, Adcyap1, Adra2a, Aplnr, Cnr2, Chrna1, Crhr2, Cysltr2, Drd1, Gabra3, Gabrg2, Gabrr2, Glra1, Gpha2, Lhcgr, Lpar6, Mtnr1a, Oprd1, Oxt, Pth2r, Ptgdr, Prss3, Sstr3, Trhr2, Ucn3*
**Osteoclast differentiation (12; *3.90E-03*):** *Traf2, Csf1r, Ikbkb, Itgb3, Mapk13, Nfatc2, Pira6, Pik3cb, Ppp3cc, Sirpa, Tgfb1, Tnf*
**Inflammatory bowel disease (8; *4.70E-03*):** *Tbx21, Il10, Il12a, Il2, Il21r, Il23a, Tgfb1, Tnf*
**Th1 and Th2 cell differentiation (9; 9*.50E-03*):** *Tbx21, Ikbkb, Il12a, Il2, Jag2, Mapk13, Notch1, Nfatc2, Ppp3cc*
**Amoebiasis (10; *1.00E-03*):** *C8g, Gnaq, Il10, Il12a, Lamc3, Nos2, Pik3cb, Plcb1, Tgfb1, Tnf*
**Tuberculosis (13; *1.90E-02*):** *Clec4e, Cebpg, Fcer1g, Cyp27b1, Il10, Il12a, Il23a, Lamp2, Mapk13, Nos2, Ppp3cc, Tgfb1, Tnf*
**Glyoxylate and dicarboxylate metabolism (5; *2.20E-02*):** *Aco1, Glul, Grhpr, Mmut, Pcca*
**Platelet activation (10; *2.40E-02*):** *Fcer1g, Adcy4, Gnai1, Gnaq, Itgb3, Mapk13, Pik3cb, Plcb1, Ptgs1, Tln2*
**Th17 cell differentiation (9; *2.50E-02*):** *T-box 21(Tbx21, Ikbkb, Il2, Il21r, Il23a, Mapk13, Nfatc2, Ppp3cc, Tgfb1*
**Retrograde endocannabinoid signaling (11; *2.80E-02*):** *Ndufa2, Adcy4, Gabra3, Gabrg2, Gabrr2, Gnai1, Gng13, Gnaq, Mapk13, Plcb1, Rims1*
**Toxoplasmosis (9; *3.90E-02*):** *Gnai1, Ikbkb, Il10, Il12a, Lamc3, Mapk13, Nos2, Tgfb1, Tnf*
**Cytokine-cytokine receptor interaction (17; *3.90E-02*):** *Acvr1b, Ccr10, Cxcr5, Csf1r, Epor, Il1rn, Il10, Il12a, Il17rc, Il17b, Il2, Il21r, Il23a, Il7, Tgfb1, Tnfrsf25, Tnf*

**Dilated cardiomyopathy (8; *3.90E-02*):** *Adcy4, Cacng2, Dag1, Itgb3, Slc8a3, Tgfb1, Tpm3, Tnf*<br><br>**Hematopoietic cell lineage (8; *3.90E-02*):** *Cd55b, Cd7, Flt3l, Csf1r, Epor, Itgb3, Il7, Tnf*<br><br>**Pyrimidine metabolism (6; *4.00E-02*):** *Cad, Cmpk2, Dck, Entpd6, Entpd8, Rrm2*<br><br>**African trypanosomiasis (5; *4.10E-02*):** *Gnaq, Il10, Il12a, Plcb1, Tnf*<br><br>**Apelin signaling pathway (10; *4.20E-02*):** *Adcy4, Aplnr, Gnai1, Gng13, Gnaq, Nos2, Plcb1, Ryr1, Serpine1, Slc8a3*<br><br>**Human T-cell leukemia virus 1 infection (15; *4.40E-02*):** *Elk4, Ranbp1, Atf2, Adcy4, Egr2, H2-Q2, Ikbkb, Il2, Msx1, Nfatc2, Pik3cb, Ppp3cc, Tln2, Tgfb1, Tnf*<br><br>**Pertussis (7; *4.50E-02*):** *Gnai1, Il10, Il12a, Il23a, Mapk13, Nos2, Tnf*<br><br>**Circadian entrainment (8; *4.70E-02*):** *Adcy4, Adcyap1, Gnai1, Gng13, Gnaq, Mtnr1a, Plcb1, Ryr1*
**Up-expressed KEGG Pathway (count; *p-value*):** *genes*
**Metabolic pathways (70; *2.80E-02*):** *Agpat1, Agpat5, Hacd1, Atic, Cd38, Ahcyl1, Setd2, St3gal2, St6galnac5, B3galnt1, Acaca, Acod1, Acsl4, Acox1, Alox12, Arsb, Camkmt, Cbr4, Cs, C1galt1, Cda, Cyp11b2, Cyp19a1, Cyp2c40, Cyp4a10, Cyp7a1, Dgkz, Dgka, Dpm2, Eno3, Fah, Galt, Gpt, Eprs, Ears2, Gsto2, Hk1, Hgd, Hyi, Idh2, Ivd, Kmt5b, Mpi, Mgat1, Mgat3, Mccc2, Mmab, Mocs2, Npr2, Ntpcr, Pank1, Prdx6, Pfkm, Pgk2, Pla2g2a, Plcd4, Plce1, Plcg1, Pmm1, Galnt2, Plod1, Ptgds, Renbp, Spr, Srr, Sgms2, Sgpl1, Srd5a2, Tktl2, Urah*<br><br>**Prolactin signaling pathway (7; *4.10E-02*):** *Sos2, Foxo3, Galt, Nfkb1, Prl6a1, Shc3, Socs1*<br><br>**HIF-1 signaling pathway (9; *4.20E-02*):** *Angpt1, Egln1, Eno3, Hk1, Nfkb1, Pfkm, Pgk2, Plcg1, Timp1*<br><br>**Glycerophospholipid metabolism (8; *5.00E-02*):** *Agpat1, Agpat5, Dgkz, Dgka, Lcat, Lypla1, Pnpla6, Pla2g2a*
